# The diagnostic value of human epididymis protein 4 for endometrial cancer is moderate

**DOI:** 10.1038/s41598-020-79960-1

**Published:** 2021-01-12

**Authors:** Jing Liu, Lili Han, Zhen Jiao

**Affiliations:** 1 Department of Gynecology, People’s Hospital of Xinjiang Uygur Autonomous Region, Urumqi, 830001 China; 2 Department of Gynecology, People’s Hospital of Xinjiang Uygur Autonomous Region, No. 1 Longquan Street, Tianshan District, Urumqi, 830001 China

**Keywords:** Gynaecological cancer, Tumour biomarkers

## Abstract

Human epididymis protein 4 (HE4) has been used as a biomarker of endometrial cancer (EC) in clinical practice. However, there remains a lack of systemic research on the critical values of HE4 for diagnosing different clinical stages and pathological types of EC. This study investigated the accuracy of human epididymis protein 4 (HE4) in the diagnosis of EC. Patients who were hospitalized for a chief complaint of abnormal vaginal hemorrhage at Xinjiang Uyghur Autonomous Region People's Hospital between 2014 and 2019 were consecutively included. Pathological biopsy confirmed the diagnosis of EC; there were a total of 136 EC patients and 127 non-EC patients. The accuracy of HE4 in the diagnosis of EC was assessed with SPSS software. The accuracy of HE4 for diagnosing different clinical stages and pathological types of EC was also explored. The critical value of HE4 for endometrial cancer was 52.40 mmol/L, with a sensitivity of 57.35% and a specificity of 76.38%. For different stages of EC, the critical value was 36.9 mmol/L, and the sensitivity and specificity were 28% and 87.39%, respectively. For different pathological types, the critical value was 30.60 mmol/L, and the sensitivity and specificity were 93.85% and 33.33%, respectively. The diagnostic value of HE4 for EC is moderate, and the serum HE4 level cannot reflect the stage and type of EC.

## Introduction

Endometrial cancer (EC) is an epithelial malignancy that arises in the endothelium lining the uterus; it is one of the three most prevalent malignancies among females (the others are cervical cancer and ovarian cancer), accounting for 20–30% of all gynecologic cancers^1^. EC is the most common gynecologic malignancy in Western countries^2^. In recent years, EC has shown an increasing incidence and younger age of onset worldwide, particularly in Asia^3,4^. According to the literature, the prognosis of early EC is satisfactory, whereas that of advanced EC is very poor^5–7^. To improve the prognosis of EC patients and thus to increase their overall survival, the key is to improve the rate of early diagnosis of EC.

Pathological biopsy serves as the gold standard for diagnosing EC, and currently, diagnostic curettage and hysteroscopic biopsy are the most frequently used procedures, and their diagnostic results are based on histological outcomes. However, pathological biopsy is often used for the preoperative diagnosis of EC. To date, scholars and researchers have proposed different methods for early EC screening, such as hysteroscopy (HS)^8^, ultrasound^9^, endometrial cytology testing^10^, magnetic resonance imaging (MRI)^11^ and tumor marker detection^12^. Among these methods, HS combined with fractional curettage is the most frequently used method for early EC screening because of its relatively high diagnostic accuracy. However, this method has some drawbacks; it is invasive, and patient tolerance is poor. Positron emission tomography-computed tomography (PET/CT) has the drawback of low sensitivity for detecting lymph node metastasis in EC^13^. In addition, CT diagnosis has a certain radiation effect, and the safety is low. Although EC can also be detected based on a Pap test^14^, this technique has not been widely applied in clinical practice. In recent years, biomarker detection has been extensively applied in the early screening of tumors. This technique has the merits of convenient performance, minimal trauma and satisfactory tolerance.

To date, a number of biomarkers for EC, such as relative telomere length in cell-free DNA^15^ and CA125^16^, have been reported. Among the various proposed biomarkers, human epididymis protein 4 (HE4) has been reported with the highest accuracy for diagnosing EC^16,17^. HE4 is a proteinase inhibitor with protective immunity, and it is an acidic micromolecular secretory protein that can be detected in serum. Currently, HE4 has been used as a biomarker of EC in clinical practice. It not only is positively expressed in normal human tissues, such as the female reproductive tract, epididymis, respiratory epithelium, breast epithelium, distal convoluted tubule of the kidney, colonic mucosa and salivary glands but also can be highly expressed in tumors, such as ovary serous carcinoma and EC^7,18^. HE4 possesses potential value in diagnosing early EC as well as in assessing the treatment outcome, judging the prognosis and monitoring relapse/migration for EC patients^19,20^. However, most of the reported functions of HE4 as an EC biomarker remain controversial. According to a meta-analysis^21^, studies on the use of serum HE4 for diagnosing EC have the following drawbacks: (1) most of the reported studies suffered from a sample selection bias, which artificially increased the sensitivity and specificity of HE4 in diagnosing EC; and 2) there remains a lack of systemic research on the critical values of HE4 for diagnosing different clinical stages and pathological types of EC.

Based on the aforementioned information, the current study assessed the accuracy of HE4 in the diagnosis of EC. The critical values of HE4 to differentiate different stages and pathological types of EC were also investigated.

## Results

### General data

A total of 263 patients were included in this study. Their mean age was 51 years, ranging from 22 to 78 years. Among these patients, 136 were confirmed to have EC, and their mean age was 54 years (ranging from 47 to 61 years). According to the FIGO staging system, 112 patients (82.3%) had stage I EC, and 24 patients (17.7%) had stage II, III, or IV EC, including 5 patients with stage II EC, 17 patients with stage III EC and 2 patients with stage IV EC. According to pathological typing, 130 patients had endometrioid adenocarcinoma (type I), and 6 had a specific pathological type (type II). In the non-EC group, 40 patients had uterine leiomyoma (age, 44.5 years (40.25, 49)), 36 patients had endometrial polyps (age, 50.5 years (42, 60.75), 22 had ovarian cysts (age, 45 years (38.75, 58)), and 29 had uterine prolapse (age, 58 years (46, 67.5)).

The EC, uterine leiomyoma, endometrial polyp, ovarian cyst, and uterine prolapse groups showed significant differences in age (χ^2^ = 33.585, *P* < 0.001; Table 1).

**Table 1:** General data (mean (Q25, Q75).

Group	Number of patients	Age
EC	136	54.00 (47.00, 61.00)
Uterine leiomyoma	40	44.50 (40.25, 49.00)^a^
Endometrial polyps	36	50.50 (42.00, 60.75)^ab^
Ovarian cysts	22	45.00 (38.75, 58.00)^a^
Anterior vaginal wall prolapse	29	58.00 (46.00, 67.50)^bcd^
χ^2^		33.585
P		< 0.001

*EC* endometrial cancer.

^a^A significant difference compared with the EC group.

^b^a significant difference compared with the uterine leiomyoma group.

^c^a significant difference compared with the endometrial polyp group.

^d^a significant difference compared with the ovarian cyst group.

### Serum HE4 level

The EC, uterine leiomyoma, endometrial polyp, ovarian cyst, and uterine prolapse groups showed significant differences in serum HE4 levels (χ^2^ = 30.049, *P* < 0.001; Table 2).

**Table 2:** Serum HE4 levels in different groups (mean (Q25, Q75)).

Group	Number of patients	Serum HE4 level	
EC	136	56.20 (41.63, 87.73)	
Uterine leiomyoma	40	38.15 (32.25, 45.93)^a^	
Endometrial polyps	36	46.85 (39.50, 58.13)^ab^	
Ovarian cysts	22	38.50 (35.73, 48.03)^ac^	
Anterior vaginal wall prolapse	29	44.10 (35.05, 56.15)^a^	

*EC* endometrial cancer.

^a^A significant difference compared with the EC group.

^b^A significant difference compared with the uterine leiomyoma group.

^c^A significant difference compared with the endometrial polyp group.

^d^A significant difference compared with the ovarian cyst group.

### Accuracy of HE4 in diagnosing EC

**Figure 1:**
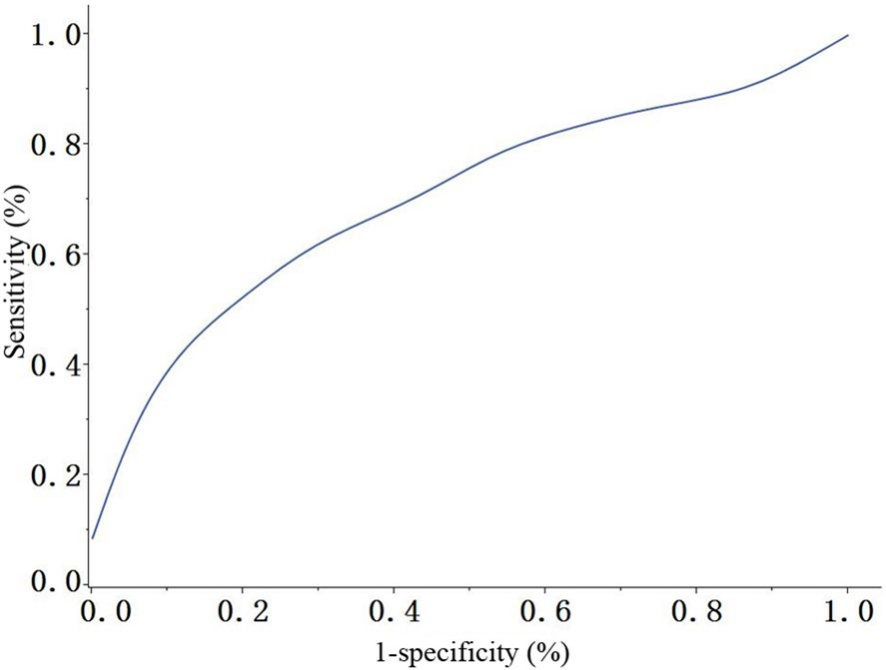
Receiver operating characteristic curve of HE4 for diagnosing EC. χ^2^ = 30.951, *P* < 0.001.

HE4 was highly expressed in the EC group. Using the non-EC group as the reference population, the obtained area under the ROC curve (AUC) of HE4 for diagnosing EC was 0.7023 (χ^2^ = 30.951, *P* < 0.001; Fig. 1; Table 3). When HE4 was 52.40 mmol/L, the Youden index reached the maximum. That is, 52.40 mmol/L was the critical value of HE4 to differentiate the EC group from the non-EC group. Based on the critical value, a four-fold table was established (Table 4).

**Table 3:** The performance indices of the HE4-based diagnostic test.

Index	Pathological biopsy (95% CI)
Sensitivity (%)	57.35 (48.59–60.70)
Specificity (%)	76.38 (67.86–83.26)
Optimal critical value	52.40 mmol/l
AUC	0.7023
Youden index	0.34
Positive predictive value (%)	72.00(62.64–80.20)
Negative predictive value (%)	62.58 (54.42–70.11)
Positive likelihood ratio	2.43 (1.72–3.43)
Negative likelihood ratio	0.56 (0.46–0.68)
Prior probability (%)	51.71
Posterior probability (%)	72.22

*AUC* area under the curve.

**Table 4:** The four-fold table of the HE4-based diagnostic test.

	Pathological biopsy	
HE4	EC patients	Non-EC patients
Positive	78	30
Negative	58	97

*EC* endometrial cancer.

### Diagnostic performance of serum HE4 for diagnosing EC

Based on the established four-fold table (Table 4), with pathological biopsy as the gold standard, the performance of serum HE4 for diagnosing EC was assessed. The results are summarized in Table 3. The sensitivity, specificity, Youden index, positive predictive value, negative predictive value, positive likelihood ratio, negative likelihood ratio, prior probability and posterior probability were 57.35% (48.59–60.70%), 76.38% (67.86–83.26%), 0.34, 72.00% (62.64–80.20%), 62.58% (54.42–70.11%), 2.43 (1.72–3.43), 0.56 (0.46–0.68), 51.71% and 72.22%, respectively.

### Critical value of serum HE4 for different clinical stages of EC

According to the FIGO staging system, 112 patients (82.3%) had stage I EC, and 24 (17.7%) had stage II, III, or IV EC, including 5 patients with stage II EC, 17 patients with stage III EC and 2 patients with stage IV EC. The critical value of HE4 to diagnostically differentiate EC at stage I from EC above stage I was 36.9 mmol/L (Table 5), with a sensitivity and specificity of 28% and 87.39%, respectively.

**Table 5:** The area under the ROC curve (AUC), optimal critical value, sensitivity and specificity of HE4 for different FIGO stages of EC.

Index	AUC	Optimal critical value	Sensitivity (%)	Specificity (%)
HE4	0.4858	36.9	28	87.39

### Comparisons of the HE4 levels and positive rates between patients at different FIGO stages

The results are summarized in Table 6. No significant differences in the positive rates and levels of HE4 were observed between the FIGO stage I group and the FIGO stage II and above group (*χ*^*2*^ = 3.700, *P* = 0.054).

**Table 6:** Comparisons of the positive rates and levels of HE4 between the FIGO stage I group and the FIGO stage II and above group (n/%).

FIGO stage	HE4-based diagnosis		*χ2*	Critical value	*P*
	Stage I	Stage II and above			
Stage I	14 (12.61%)	97 (87.39%)	3.700	36.9	0.054
Stage II and above	7 (28.00%)	18 (72.00%)			

### Critical value of HE4 in the diagnosis of different types of EC

According to pathological typing, 130 patients had endometrioid adenocarcinoma (type I), and 6 a specific pathological type (type II) in this study. The critical value of HE4 to differentiate type I from type II was 30.60 mmol/L (Table 7). The positive rate and level of HE4 in the type I group did not show significant differences compared with those in the type II group (*P* > 0.05; Table 8).

**Table 7:** The area under the ROC curve (AUC), optimal critical value, sensitivity and specificity of HE4 for different pathological types of EC.

Index	AUC	Optimal critical value	Sensitivity (%)	Specificity (%)
HE4	0.5244	30.60	93.85	33.33

**Table 8:** Comparisons of the positive rates and levels of HE4 between the type I group and the type II group (n/%).

HE4-based	Pathological type		P
	Type I	Type II	
Type I	8 (80.00)	2 (20.00)	0.054*
Type II	122 (96.83)	4 (3.17)	

*Fisher’s exact probability.

## Discussion

EC is the most common malignancy of the female reproductive system. In this study, the diagnostic value of serum HE4 for EC was systemically investigated based on the suspected case inclusion method. The critical values of HE4 for different stages and pathological types of EC were also calculated.

To date, only a few studies have been reported on the critical value of HE4 in the diagnosis of EC. Even among this small number of studies, the reported critical values differed greatly. For example, the reported critical value by Ma et al. was 141.5 mmol/L^22^, whereas that reported by Fang et al. was 45.52 mmol/L^23^. In this study, for the first time, suspected cases were included, which were used to calculate and obtain the exact AUC value of HE4 in diagnosing EC (0.7023; χ^2^ = 30.951, *P* < 0.001). When the HE4 level was 52.40 mmol/L, the Youden index reached the maximum value, and the calculated sensitivity and specificity were 57.35% and 76.38%, respectively. In addition, this study showed that the serum HE4 level in patients with EC significantly differed from that in those with uterine leiomyoma, ovarian cysts, endometrial polyps and uterine prolapse. These findings indicate that HE4 is highly expressed in EC and suggest that serum HE4 has a high, promising diagnostic potential for EC.

The FIGO clinical stage is closely associated with the prognosis of EC patients^24,25^. In this study, 136 EC patients were included, including 112 patients with stage I EC and 24 patients with stage II, III, or IV EC. We found that the optical critical value of HE4 for stage differentiation was 36.9 mmol/L, at which the sensitivity and specificity were 28% and 87.39%, respectively. In addition, our study showed that patients with stage I EC and those with stage II, III, or IV EC did not show significant differences in the positive rate and level of HE4. These findings contrasted with those reported in the literature; HE4 has been reported to be an independent assessment factor for the prognosis of EC, and its level is closely associated with FIGO stage, cell differentiation, myometrial infiltration and lymph node migration^26^; the HE4 level is associated with the FIGO stage, and it is significantly altered after treatment^16,27^. Presumably, the reasons for these inconsistencies were that this study included a small sample size and that it was a retrospective study.

According to the WHO classification criteria, tumors of female reproductive organs can be pathologically divided into type I and type II tumors, and the prognoses of these two types vary greatly. Zhao et al. detected HE4 levels in 80 patients with different pathological types of EC and found that the preoperative HE4 level was positively correlated with the extent and depth of EC infiltration as well as the lymph node migration status^28^. The serum HE4 level is closely associated with the pathological type of EC, depth of myometrial infiltration, size of the tumor and progression of the disease^29^. Among the 136 patients included in this study, 130 patients had type I EC, and 6 patients had type II EC. The critical value of HE4 for the differentiation of different EC types was 30.6 mmol/L, with a sensitivity and specificity of 93.85% and 33.33%, respectively. In addition, the type I group and the type II group did not show significant differences in the positive rate and level of HE4. HE4 is positively expressed in EC tissues^11^, but its level in serum has not been associated with the pathological type of EC^10,30–32^. Our findings were consistent with those reported in the literature.

HE4 overexpression has been found in patients with EC as well as in patients with transitional cell carcinoma, pulmonary adenocarcinoma, breast cancer, pancreatic cancer and ovarian cancer^10,33^. Serum HE4 levels can be used as a prognostic indicator for EC patients^34^. A high HE4 level is an independent prognostic factor for patients with poorly differentiated EC; this finding may provide a new idea for clinicians to take active adjunctive measures for EC patients in a critical condition^32^. In this study, our findings indicated that HE4 had a high diagnostic value for EC. For women with high-risk factors for EC, early serum HE4 detection may increase the diagnostic rate of early EC and therefore reduce the rate of missed diagnosis.

This study had some limitations. First, the sample size of this study was limited, and the nature of this study was retrospective, which might cause biases in the statistical analyses. To validate the outcomes of this study, large-scale, multi-centered, longitudinal and prospective studies need to be conducted in the future. Second, the follow-up time, particularly for the patients included in 2019, was short. Therefore, the correlations of HE4 levels with lymph node migration in patients with EC and EC relapse were not analyzed, which constituted another major limitation of this study. To overcome this limitation, longer follow-ups need to be carried out in the future to comprehensively evaluate the value of serum HE4 in screening and monitoring for EC.

In conclusion, serum HE4 has a moderate diagnostic value for EC, and its level may not reflect the clinical stage and pathological type of EC.

## Materials and methods

### Data collection

Clinical records of the suspected EC patients who were hospitalized at Xinjiang Uyghur Autonomous Region People's Hospital between 2014 and 2019 were consecutively collected and retrospectively analyzed. All patients had a chief complaint of abnormal vaginal hemorrhage, and HE4 was detected using enzyme-linked immunosorbent assays (ELISA; the kit was purchased from CanAg Diagnostics AB (Sweden), and the I2000SR ELISA instrument was a product of Abbort (US)). Data of the patients with and without EC according to pathological biopsy were collected, and the corresponding patient number and HE4 values were separately recorded. A third party was invited to check data to guarantee accuracy. The inclusion criteria were as follows: (1) abnormal vaginal hemorrhage as the chief complaint; (2) availability of the HE4 value; (3) underwent EC pathological biopsy; and (4) underwent EC surgery. Patients who met any of the following criteria were excluded from the current study: (1) renal insufficiency; (2) other diseases, such as ovarian cancer and lung cancer, that can lead to an increased HE4 level; and (3) incomplete clinical data.

The protocols of this study were approved by the Ethics Committee of Xinjiang Uyghur Autonomous Region People's Hospital, and all methods were performed in accordance with the Declaration of Helsinki. Written informed consent for research purposes was obtained from each participant.

### Observational indices and methods

In this study, EC was staged according to the latest criteria emended by the Federation Internationale de Gynecologie et d'Obstetrique (FIGO) in 2009. In addition, according to the World Health Organization (WHO) classification criteria for female reproductive tumors, EC was divided into the estrogen-dependent type (type I) and the estrogen-independent type (type II). In the former, a typical histological type is endometrioid adenocarcinoma, which reacts with estrogen treatment. In the latter, the typical histological types include serous carcinoma and clear cell carcinoma, which have no response to estrogen treatment. Data were statistically calculated and then input into Excel.

### Statistical analysis

Data were processed with SPSS 22.0. Measurement data in a normal distribution are presented as the mean ± standard deviation (x ± s), and Student’s *t*-test was used to compare data between groups. Abnormally distributed measurement data were presented as the median and interquartile range, and the Wilcoxon rank-sum test was used for comparisons. A value of P ≤ 0.05 was considered to be statistically significant. A receiver operating characteristic (ROC) curve was plotted with SPSS 22.0. Based on the Youden index, the cutoff value of HE4 was determined. Values above the critical value were considered positive, and those below the value were considered negative. Then, a four-fold table of the diagnostic test was established. The sensitivity, specificity, predictive values, likelihood values and Youden index of HE4 were calculated to assess its accuracy (sensitivity and specificity) in diagnosing EC.
